# Membrane Fouling Due to Protein—Polysaccharide Mixtures in Dead-End Ultrafiltration; the Effect of Permeation Flux on Fouling Resistance

**DOI:** 10.3390/membranes9020021

**Published:** 2019-02-01

**Authors:** Dimitrios Sioutopoulos, Anastasios Karabelas, Vasileios Mappas

**Affiliations:** 1 Chemical Process and Energy Resources Institute, Centre for Research and Technology-Hellas, 6th km Charilaou-Thermi Road, 57001 Thermi-Thessaloniki, Greece; sioutop@cperi.certh.gr; 2 Department of Chemical Engineering, Aristotle University of Thessaloniki, 54623 Thessaloniki, Greece; vm379@cam.ac.uk

**Keywords:** dead-end ultrafiltration, combined polysaccharide-protein fouling, specific fouling resistance, correlation with permeate flux, compressibility effects

## Abstract

Significant gaps exist in our knowledge of ultrafiltration (UF) membrane fouling, due to mixtures of poly-saccharides and proteins, despite a fair amount of related research. To get new insights into fouling layer characteristics, experiments were performed under constant-flux, within the range of practical interest (15–90 L/m^2^h), with typical polysaccharides (sodium alginate, SA), proteins (bovine serum albumin, BSA) as well as their mixtures in various proportions (1:3, 1:1, 3:1), and total organic matter concentration of 30 mg/L. The feed-water salinity and calcium ion concentration were 2000 mg/L NaCl and 2 mM, respectively. The temporal evolution of such fouling layers on flat-sheet membranes was monitored by recording the trans-membrane pressure variation. The results show that the specific fouling resistance α is strongly affected by flux, and the fouling propensity of polysaccharide-protein mixtures is significantly enhanced compared to single foulants, i.e., when BSA and SA are alone. The fouling layers are compressible and their resistance α tends to increase with the mass ratio of alginate in the mixture, particularly at high fluxes. To quantify these effects, correlations are presented of the initial fouling resistance α_i_ with permeate flux J and of the evolution of α. R&D priorities are suggested on this topic of mixed foulants.

## 1. Introduction

Membrane fouling by dissolved organic matter is an inherent problem in the Ultrafiltration (UF) treatment of various effluents, including the pre-treatment of feed-waters to RO/NF plants. Proteins and polysaccharides are ever-present in effluents to be upgraded [1,2], particularly in those originating from the food-processing industry and many other effluents/waters that are contaminated due to biological activity or being biologically treated by MBR [3,4,5,6,7]. In the latter very common cases, the usual contaminants are due to the Extracellular Polymeric Substances (EPS) that are mainly comprised of polysaccharides, proteins, natural organic matter and lipids—the former two make up the greatest proportion [3,4,6,7,8,9,10].

A significant amount of research has been performed on the fouling characteristics of UF membranes by polysaccharides and proteins [1,2], mainly when they are present alone in the solution/feed-water to be treated. However, the studies on UF membrane fouling by mixtures of polysaccharides and proteins are much less common, with significant knowledge gaps, that have a negative impact on the design and operation of UF systems (e.g., reference [11]). In most studies, Bovine Serum Albumin (BSA) and Sodium Alginate (SA) have been employed, which are typical of proteins and polysaccharides encountered in practice. For this reason, these organic compounds have also been used in the present study.

In the case of mixed foulants as the present ones (BSA and SA), one may recognize three main issues regarding fouling of membranes: (a) The physico-chemical interaction in the fluid bulk of the different (macro-) molecules, as a function of the ionic environment and other properties (pH, temperature, etc.); (b) the interaction with the membrane surface of the particular organic molecules and/or their complexes and agglomerates; (c) the evolution of the membrane fouling deposits (commonly in the form of a layer comprising the organic species) in the dynamic environment shaped mainly by the transmembrane pressure and the resulting flux. As suggested by the following brief review, regarding UF membrane fouling, most of the published work deals with topics (a) and partly (b), whereas rather limited work is available on combined SA-BSA fouling evolution.

*SA and BSA, alone in solution*, tend to interact with the ionic environment. For dilute aqueous solutions (representative of waters to be treated), the most well known type is the complexation of SA macromolecules with cations (mainly Ca and Mg) [12,13]. At higher concentrations (as in the case of boundary/fouling layers at the membrane surface) SA-Ca gels develop with pronounced viscoelastic properties [14]. BSA alone also tends to form gels, apparently comprising a continuous network that is affected by the ionic environment.

A fair amount of work has been reported on the *physico-chemical interactions of BSA with SA in solution*, in the presence of cations that tend to promote formation of complexes of those macro-molecules, although knowledge gaps still exist. Rather strong non-covalent interactions have been identified (e.g., reference [15]) that depend on the solution pH; in fact, at sufficiently low pH below the protein iso-electric point, positively charged regions of protein molecules form fairly strong bonds with the negatively charged carboxyl groups of alginate [16]. Under these conditions the highest membrane fouling rates were measured. Neiser et al. [17], by measuring the Young modulus, observed that the BSA/Ca-alginate gels were stronger that those of BSA/Na-alginate as well as those of pure BSA gels. The effect of Ca was dominant in both pure Alginate and BSA/Alginate gels.

Regarding the *interaction of BSA and SA foulants with membranes*, most of the reported work has been performed with RO membranes [18]. Wu et al. [19] have studied the effect of specific surface functional groups on RO membrane fouling by alginate under conditions relevant to seawater desalination. Ang and Elimelech [20] report that RO-membrane fouling by BSA is enhanced by increased calcium concentration and solution pH at the BSA isoelectric point (pH 4.7). Similar results were obtained by Mo et al. [21]. Moreover, BSA fouling of RO membranes is significantly enhanced in the presence of alginate. Surface roughness, zeta potential, and hydrophilicity play a role [22]. In the presence of Ca-ions, alginates foul RO membranes faster than BSA, whereas BSA/SA synergistic effects are also observed. Ang et al. [23] have also measured strong intermolecular adhesion forces between alginate and BSA molecules (in the bulk) in the presence of Ca^2+^ that result in a rather ‘compact’ conformation of these aggregates/foulants, which tend to form a “tighter”/compact fouling layer on RO membrane, compared to that resulting from a solution of Ca-alginate alone. The latter tends to form extended gel networks that are favored by the macro-molecular structure of alginates [12,13]. Susanto et al. [2] using PES UF membranes observed the interaction effects of polysaccharides with proteins that led to mixed fouling layers with stronger reduction of flux than those from individual foulants under the same conditions; the effect of pH and membrane surface properties on fouling was also examined in that work.

Relevant studies on UF membrane fouling have been carried out in the authors’ laboratory, mostly with alginates [24,25,26,27,28,29]. The *specific fouling resistance α* has been used for data interpretation and correlation with the permeate flux J [30,31]. Under constant flux J mode of UF filtration, the non-linear increase of pressure drop across the fouling layer, ΔP_c_, with time (and the concomitant increase of resistance α) was indicative of strong compressibility effects on the evolving alginate fouling layer [31]. In efforts to quantify such effects for dead-end UF filtration, a generalized correlation was suggested of resistance α data, taken with SA in low salinity water. The approach taken in these studies is considered appropriate for interpreting similar data for mixtures of foulants such as BSA and SA.

The above overview of the literature suggests that the research so far has improved our understanding on UF membrane fouling by mixtures of BSA and SA. However, a systematic analysis of fouling data from such studies, in terms of a representative fouling parameter (and correlation thereof with key process parameters) seems to be missing. Therefore, this study aims to determine the effect of main parameters (i.e., permeation flux J, pressure difference due to fouling) and foulants composition, on the specific fouling resistance α, which is considered as the most representative parameter of membrane fouling [26,27,28]. An additional objective is to obtain correlations of the fouling resistance α data with the key process parameter (flux J) in an effort to maximize the usefulness of the work for practical applications. In the ensuing sections, the theoretical background for data interpretation is outlined first, the experimental conditions are presented next, followed by data analysis and correlation. The closing comments outline challenges and recommendations for future R&D.

## 2. Theoretical Background

Membrane filtration under constant pressure or constant permeate flux J is described by a form of the Darcy equation

(1) $$J=\frac{1}{A}\frac{\mathrm{dV}}{\mathrm{dt}}=\frac{\left[\Delta P\right]}{{\mu [R}_{m}{+R}_{c}]}$$

where R_m_ and R_c_ are the clean membrane and fouling resistance, respectively. An initial trans-membrane pressure (TMP), ${\left[\Delta P\right]}_{o}{=\mu R}_{m}J$, can be determined at time t = 0, i.e., before membrane fouling starts. Considering that a layer formation is the dominant fouling mechanism and assuming that R_m_ remains constant, the pressure drop due to fouling, ΔP_c_, is determined as follows by monitoring the TMP:

(2) $$[\Delta P]-{\left[\Delta P\right]}_{o}\equiv \Delta P_{c}$$

Combining Equations (1) and (2) one obtains

(3) $$[\Delta P]=\mu JR_{m}+\mu JR_{c}={\left[\Delta P\right]}_{o}+\mu JR_{c}$$

Starting with a clean membrane, the resistance R_c_ is related to specific fouling resistance α, i.e.,

(4) $$R_{c}=\alpha \cdot m=\frac{\alpha \cdot C\cdot V}{A}$$

Considering that in practice the effective foulant concentration C (for fouling layer formation) may be unknown, it is common to employ the quantity I = (α·C) designated as the fouling index. For ultra-filtration of solutions of organic macro-molecules, the rejection of such species is generally high but may not be 100%. Therefore, the effective concentration C, used in Equation (4), is obtained by multiplying the known bulk concentration with an experimentally determined rejection factor.

In dealing with the *initial phase* of membrane fouling, a “clean-membrane” resistance R_m_ is considered. Thus, fouling resistance α can be determined by combining Equations (1)–(4),

(5) $$\frac{\Delta P_{c}}{{\left[\Delta P\right]}_{o}}=\frac{\alpha \cdot C}{R_{m}}\cdot \frac{V}{A}=\frac{\alpha C}{R_{m}}J\cdot t=\frac{I\cdot J}{R_{m}}\cdot t$$

In the initial phase of membrane filtration under constant flux J and for constant R_m_, by recording ΔP_c_ as a function of time, one can directly compute the temporal variation of I and (knowing the concentration C) determine the temporal evolution of resistance α. Equation (5) is applicable to dead-end ultra-filtration (of interest here) under constant flux J and fluid properties. An alternative form of Equation (5), substituting [ΔP_o_] from Equation (1), is as follows

(6) $$\Delta P_{c}=\mu \cdot \alpha \cdot C\cdot J^{2}\cdot t$$

## 3. Materials and Methods

### 3.1. Organic Foulants-Feed Solutions

Sodium alginate (A-2158, Sigma-Aldrich, St. Louis, MO, USA) extracted from brown algae was used in this study. This sodium alginate (SA) product is reported [32] to have molecular weight in the range of 12–80 kDa, with a ratio of mannuronic (M) to guluronic (G) blocks (M/G) = 1.67. A fresh concentrated sodium alginate solution was prepared a day prior to experiment by dissolving the appropriate amount of SA in deionized water and stirring overnight. Bovine serum albumin (A7906, Sigma-Aldrich, St. Louis, MO, USA) was used. Based on the manufacturer, BSA is a single polypeptide chain consisting of about 583 amino acid residues and no carbohydrates. At pH 5–7 it contains 17 intra-chain disulfide bridges and 1 sulfhydryl group, with molecular weight 66,430 Da [33].

First, concentrated stock solutions of the polysaccharide (1 g/L), proteins (1 g/L), NaCl (75 g/L) and CaCl_2_·2H_2_O (29.4 g/L) were prepared by dissolving appropriate amounts of reagents in distilled water. Tap water was used with salinity of approximately 500 mg/L (equivalent NaCl) and a corresponding calcium concentration of 1mM. By introducing concentrated solutions of NaCl and CaCl_2_·2H_2_O, final feedwater salinity and calcium concentration were adjusted at 2000 mg/L and 2 mM, respectively. Other chemicals used in the fouling experiments included HCl and NaOH to adjust the feed stream pH to 7.0. All reagents employed were analytical grade. The composition of the concentrated stock solutions employed in the tests are summarized in Table S1, Supplementary Materials. The total concentration of organic species in the feed-water was 30 mg L^−1^, which is typical of foulants concentration in feed-waters treated by UF membranes. To examine the effect of feed-fluid SA:BSA mass ratio on membrane fouling, five different mass ratios of SA over BSA were examined; i.e., 100% SA, 75% SA:25% BSA, 50% SA:50% BSA, 25% SA:75% BSA, 100% BSA.

According to protocol, concentrated solutions of SA and BSA were mixed in appropriate amounts, to a total volume of 30 mL, and continuously agitated for 10 min. In parallel, 945 mL of tap water, 5 mL of stock CaCl_2_ solution and 20 mL of stock NaCl were mixed in a vessel (1 L) and agitated (250 rpm) for 10 min. Next, the solution with the organic foulants was slowly poured into the salt solution under high agitation (500 rpm), for final feedwater solution preparation. Another protocol was employed for feed-water preparation to check whether the sequence of mixing the various compounds had a significant impact on feed-solution characteristics and in turn on membrane fouling. In the second protocol, the same type of feed stock solutions and the same volumes were used. However, the mixing sequence of stock solutions was different; i.e., each organic fouling compound (SA and BSA) was directly mixed with the solution containing ionic species (sodium chloride and calcium chloride). Thus, a sodium alginate solution and a bovine serum albumin solution were separately prepared. After stirring (for 10 min), the individual solutions were mixed for final feed-water preparation.

### 3.2. Analytical Methods

For determination of the carbohydrate concentration, the Phenol–Sulfuric Acid reaction method described by DuBois et al. [34] was employed. Thus, alginate concentration was determined by a UV–vis double beam spectrophotometer (Shimadzu, UV-1700, Kyoto, Japan) at a wavelength of 490 nm. BSA concentration was also determined spectrophotometrically by the Bradford method, based on binding of Coomassie Blue G250 dye to proteins, by measuring solution absorbance at 595 nm. Other monitored parameters included pH and conductivity, by using a WTW inoLab multi-parameter analyzer (pH/ION/Cond 750) coupled with a WTW pH Electrode (SenTix81) and a WTW conductivity electrode (TetraCon 325). These measurements were carried out according to standard methods for the examination of water and wastewater [35].

### 3.3. Membrane Type

A hydrophilic polyacrylonitrile (PAN) ultrafiltration flat-sheet membrane was employed with a reported MWCO of 20 kDa (AMI™ Applied Membranes Inc. Vista, CA, USA). This UF membrane was selected for its hydrophilicity and reduced sensitivity to compaction, as reported elsewhere [36]. Prior to filtration, UF membranes were thoroughly rinsed with water to remove the conservation chemicals (sodium meta-bisulphite, food grade). Membrane specimens used in the experiments had a clean water resistance varying within a relatively narrow range; i.e., R_m_ = 1.13 × 10^12^ (±1.50 × 10^11^) m^−1^. Figure S1 in Supplementary Materials depicts the distribution of the R_m_ values. A new membrane coupon was employed in each fouling test and its R_m_ value was determined by conducting a test with distilled water.

### 3.4. Experimental Set-Up and Procedures

The experimental set-up employed in the experiments under *constant flux* is presented in detail elsewhere [27]. It includes a SEPA-ST model cylindrical test cell (Osmonics Inc., Minnetonka, MN, USA) accommodating a membrane disk of diameter 4.0 cm (resting on a porous support), and active filtration area of 12.7 cm^2^. All tests in this study were performed with no fluid agitation. To maintain nearly constant feed-fluid conditions, the filtration cell was kept completely filled with test liquid (V ~ 330 mL); constant flux filtration was controlled through a piston pump (Fluid Metering Inc., Syosset, NY, USA), connected with a feed-fluid vessel (under agitation). The prevailing trans-membrane pressure (TMP) was monitored by a pressure transducer (Cole-Palmer Instr. Co., Vernon Hills, IL, USA) connected at the cell inlet. The permeate flux was determined by measuring the permeate volume with an electronic balance (Mettler Toledo PB3001), interfaced with a computer for continuous data acquisition.

The experimental procedure for constant flux UF fouling experiments comprises the following steps. First, the membrane coupon (stored in 0.75% sodium bisulfite solution) is rinsed with distilled water so that preservation agents are removed and then placed in the filtration test section. Next, the test cell is filled with distilled water and the pump volumetric flow rate is adjusted to maintain a permeate flux of 60 L/m^2^h until 100 mL of permeate is collected. In general, the membrane conditioning procedure ensures efficient removal of UF membrane preservatives and stable membrane performance (by effecting some compaction), thus allowing accurate determination of intrinsic clean membrane resistance at the desired permeate flux value. After the conditioning phase, the filtration test took place with a particular test solution at constant permeate flux until a permeate volume of ~60 mL was obtained. Under such conditions, the duration of each fouling test depended on the flux but the deposited organic mass on the membrane surface was approx. 1 g/m^2^.

Foulants rejection by the UF membrane was calculated as follows, based on the organic matter concentration of feed-water C_f_ and permeate C_p_. It is noted that only the composite concentration of permeate stream C_p_ was determined; however, it is expected that the instantaneous C_p_ would not substantially change during filtration.

(7) $$\%\;\mathrm{Rejection}=\left(1-\frac{C_{p}}{C_{f}}\right)\times \;100$$

## 4. Results and Discussion

### 4.1. Temporal Variation of Pressure Drop Due to Fouling ΔP_c_

At the beginning of an organic fouling experiment under constant permeate flux, a short transient period (1–2 min) was observed, which is required to overcome the specific pressure drop due to membrane resistance, ΔP_o_, at the specific flow conditions. ΔP_o_ was determined after the conditioning test, performed prior to each fouling test. In such tests distilled water was employed and the system was operated for sufficient time until the applied pressure attained a steady state value, representing the pressure drop across the membrane for a specific permeate flux. Pressure drop values due to clean membrane resistance ΔP_o_ for each test are listed in Tables S2 and S3 (Supplementary Materials). For each test, the variation of pressure drop across the fouling layer developing on the membrane surface, ΔP_c_, is accurately determined by subtracting the initial value ΔP_o_ from the recorded applied pressure variation. More details on the ΔP_c_ determination can be found in previous publications [29,31].

**Figure 1:**
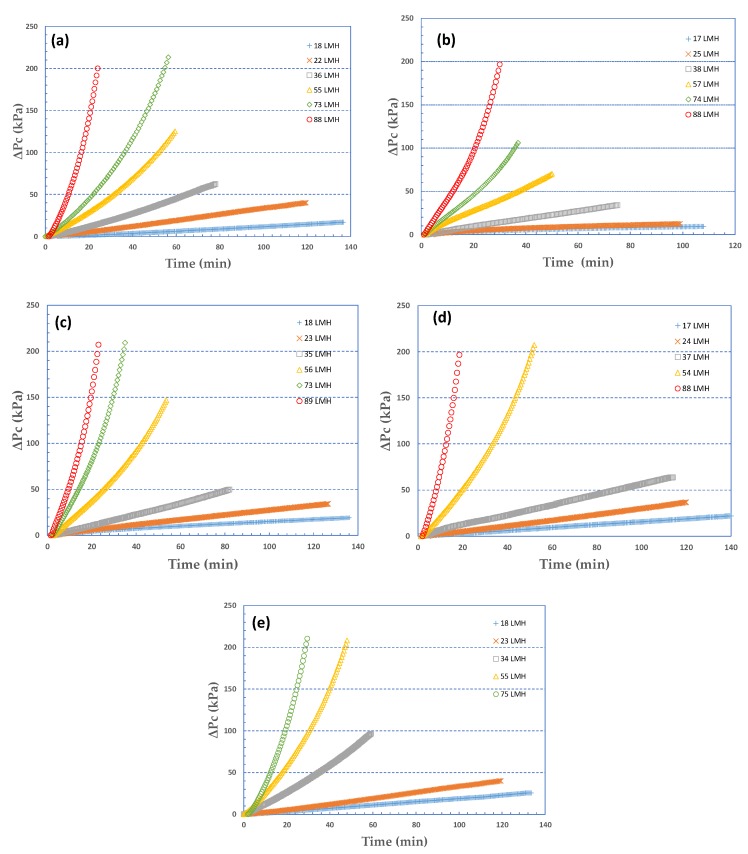
Temporal variation of pressure drop due to fouling, ΔP_c_, for various constant fluxes J and five feed-fluid foulants composition. Fouling species: (**a**) 100% SA, (**b**) 100% BSA, (**c**) 25% SA-75% BSA, (**d**) 50% SA-50% BSA, (**e**) 75% SA-25% BSA.

In Figure 1, the temporal variation is depicted of the pressure drop due to fouling ΔP_c_, for the five sets of organic fouling experiments, that correspond to foulants: 100% Sodium Alginate (SA), 100% Bovine Serum Albumin (BSA) (Figure 1a,b) and three SA-BSA mixtures in varying proportions 1:3, 1:1, 3:1 (Figure 1c,d,e). In all cases total organic matter concentration was 30 mg/L, while permeate flux was maintained constant throughout the fouling tests. It is noted that tests were carried out while pursuing similar deposited foulant-mass (approx. 1 g/m^2^) on the membrane surface to facilitate comparison of the fouling layer resistance behavior. The range of constant fluxes investigated was 15–90 L/m^2^h. Therefore, the duration of the fouling tests (listed in Table S2 Supplementary Materials) was different depending on flux; i.e., experiments conducted at high permeate flux level were run for a shorter time period compared to those at lower flux.

**Figure 2:**
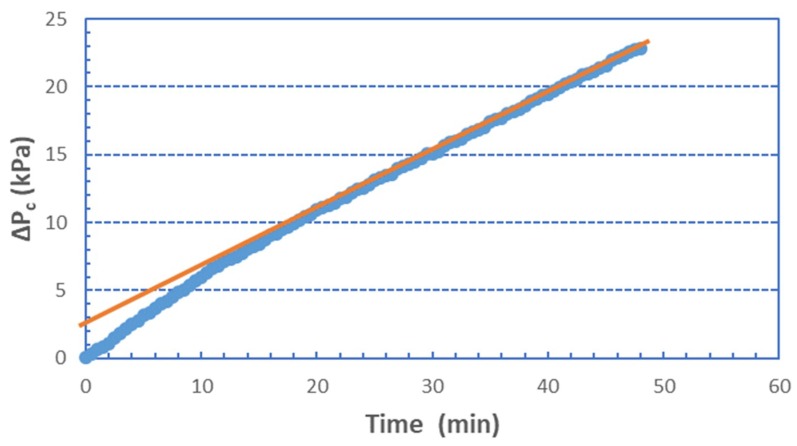
Pressure drop ΔP_c_ across 100% BSA fouling layer. Permeate flux J = 34 L/m^2^h.

Upon inspection of the graphs in Figure 1, the following observations are made:

(a) In all cases of different foulants composition, at low fluxes (J < 40 L/m^2^h), there is almost linear temporal variation of ΔP_c_ which is indicative of nearly constant specific fouling resistance α. At greater fluxes, the concave shape is typical of fouling layer compressibility effects, already observed in previous studies [29].
(b) The resistance to liquid permeation due to fouling R_c_ is high even at relatively low fluxes; this is also shown in Table S2, where values of intrinsic membrane resistance R_m_ are included for comparison. At the higher fluxes (J > ~40 L/m^2^h), fouling resistance R_c_ reaches high values, i.e., an order of magnitude greater than R_m_ within the time period of present tests.
(c) Pure BSA, and rich in BSA, foulants (Figure 1b–d) particularly at low fluxes, exhibit initially (for a few minutes) a non-linear (convex) ΔP_c_ increase. Figure 2, at greater resolution, better shows this trend, which is due to the unclear mechanism of incipient membrane fouling. One might hypothesize that there is a partial pore blocking and gradual membrane-surface coverage by the relatively compact BSA molecules and agglomerates. Beyond this initial period, a linear ΔP_c_ variation (*at the smaller fluxes*) is a likely manifestation of a coherent fouling layer formation and further growth due to organic mass deposition. As the proportion of SA in the foulant mixture is increased, this trend disappears, the alginate gel matrix apparently dominates, and the ΔP_c_ profiles exhibit linearity throughout the test period.

**Figure 3:**
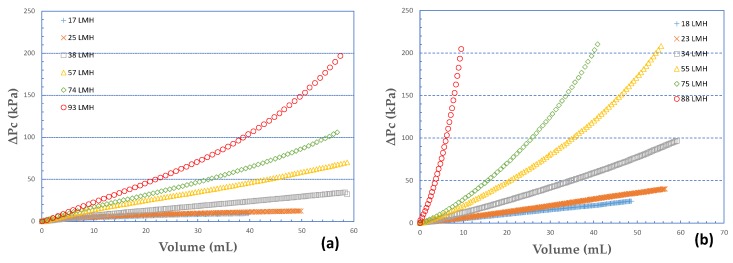
Variation of pressure drop due to fouling layer ΔP_c_ versus permeate volume. Fouling species: (**a**) 100% ΒSA, (**b**) 75% SA-25% BSA.

The strong effect of permeate flux J on the fouling layer characteristics is clearly evident in Figure 3a,b where ΔP_c_ is plotted versus the respective permeate volume for two cases, i.e., 100% BSA, 75% SA-25% BSA. In these graphs, a certain nearly constant mass of foulant deposit corresponds to a specific value of permeate volume, regardless of the flux prevailing in the tests. The validity of this statement is supported by the nearly constant high rejection of organic matter, irrespective of foulant composition, as will be subsequently shown. The graphs clearly suggest that the increased permeate flux J and the concomitant higher permeate-fluid drag forces tend to affect the structure of the fouling layer and reduce its permeability. These trends, already observed in a previous study with 100% alginate foulants at smaller concentration [29], will be subsequently examined and quantified.

### 4.2. Characteristics of Specific Fouling Resistance α

**Figure 4:**
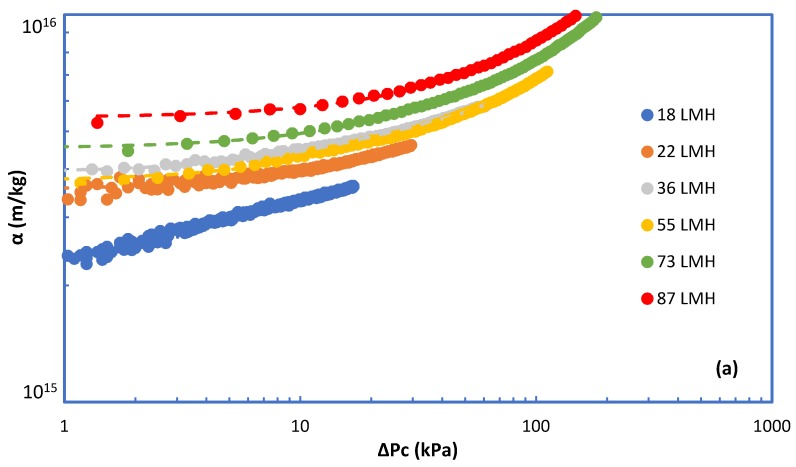

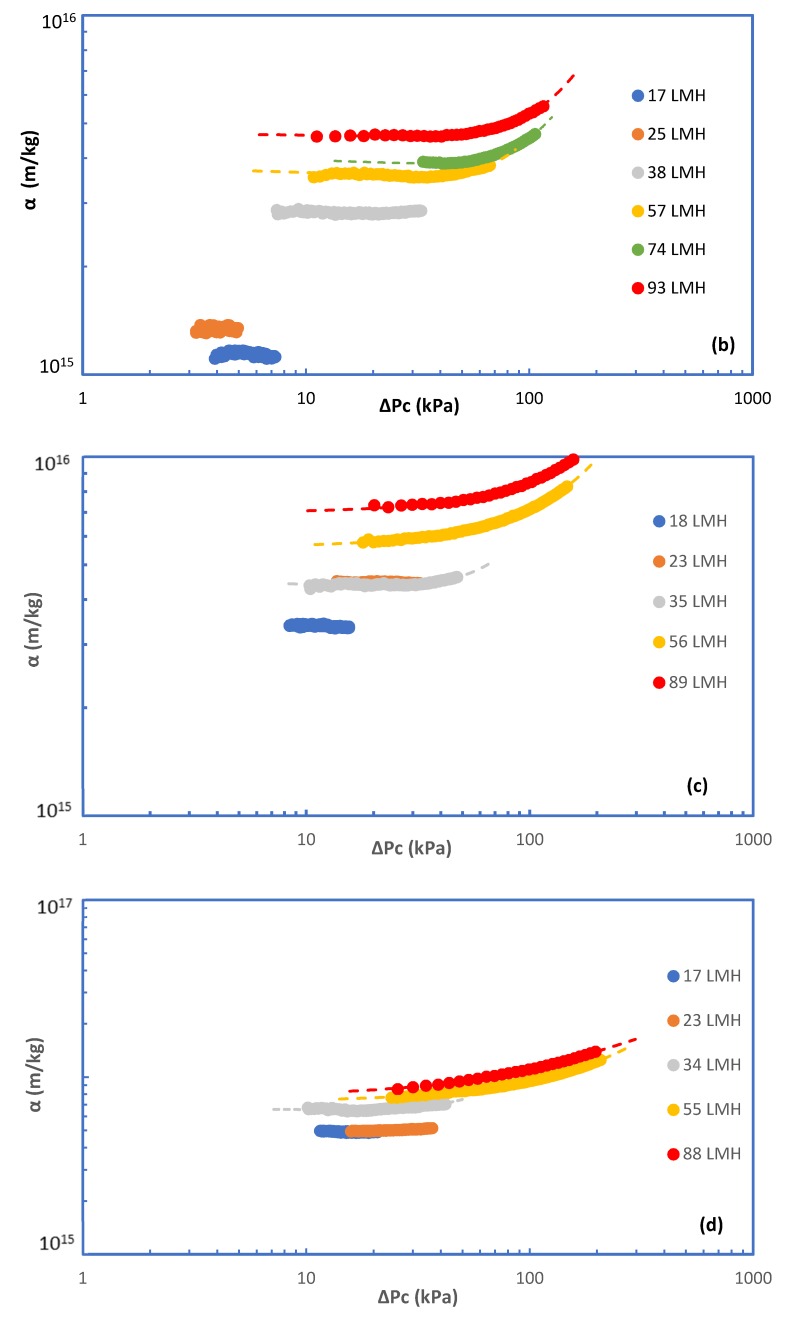

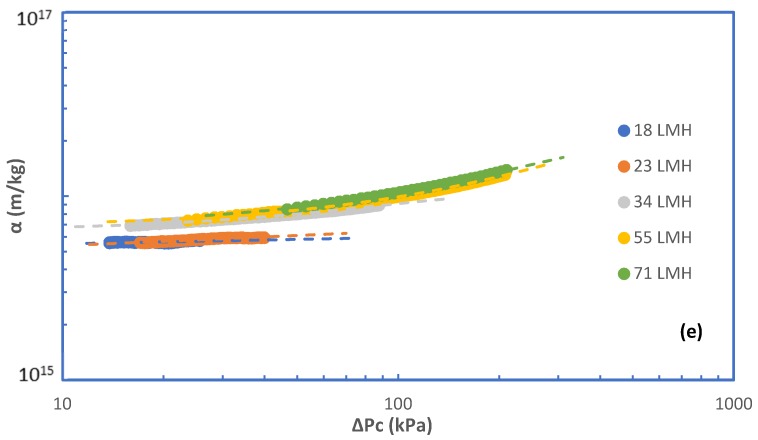
Specific cake resistance α versus pressure drop due to fouling ΔP_c_. Fouling species: (**a**) 100% SA, (**b**) 100% BSA, (**c**) 25% SA-75% BSA, (**d**) 50% SA-50% BSA, (**e**) 75% SA-25% BSA.

Figure 4a–e depict the variation of specific fouling resistance α with ΔP_c_ for all cases investigated. These results are illuminating, showing the effects of foulant composition and of flux J on the evolution of fouling resistance α. Almost all the α-profiles show a nearly constant initial resistance α_i_ corresponding to rather small ΔP_c_ values. For relatively small fluxes, regardless of foulants composition, α tends to remain constant which is indicative of an invariable fouling layer structure, despite its increasing thickness by mass addition/deposition. However, at the higher fluxes, (J > 30 L/m^2·^h), the non-linear α evolution suggests fouling layer structural changes caused by the increased fluid drag and the concomitant compressive stresses. The rheological characteristics of similar fouling layers developing on RO desalination membranes [32] and on MBR–UF membranes [37], which were experimentally determined in the authors laboratory, facilitate explanation of these treads. Indeed, these and other studies have shown that poly-saccharide-based fouling layers are viscoelastic “materials” that are deformable and strongly affected by permeation drag.

**Figure 5:**
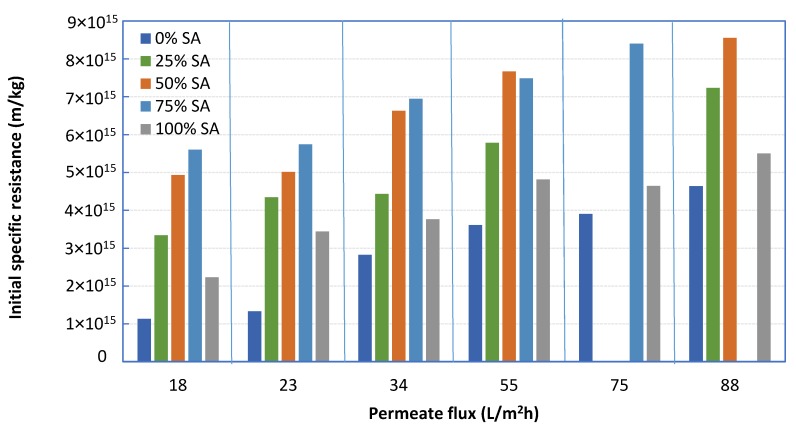
Initial fouling resistance α_i_ values, for various foulant compositions and fluxes.

The effect of *foulant composition* on initial fouling resistance α_i_ is also quite pronounced, as already reported in previous literature studies [2,23]. As shown in Figure 5, the fouling resistance of 100% BSA solution is significantly smaller than that of 100% SA, for all fluxes examined. However, by increasing the percentage of SA (in mixtures with BSA), the fouling layer resistance tends to systematically and significantly increase above that of either BSA or SA alone in solution. In fact, the greatest α values were obtained with the 25% BSA-75% SA mixture for all constant flux tests (Figure 5). These data are in qualitative accord with test-results reported in the literature where similar mixtures were employed [2,23]. It is noted that missing data in Figure 5 were excluded after the experimental campaign, due to inconsistencies attributed to equipment malfunctioning. The percentage error in determining the resistance α_i_ is estimated to be within ±5%.

### 4.3. Correlation of Fouling Resistance α Data-Comparison with Previous Studies

As in a previous study [29], an empirical expression, of the following form, will be used for correlating specific resistance α data:

(8) $$\alpha =\alpha_{i}[1+\frac{\Delta \mathrm{Pc}}{P_{o}}{]}^{n}$$

Here P_o_ and n are two parameters, representing a “reference pressure” and a “compressibility index”, respectively. In the case of incompressible fouling layers, usually encountered at relatively low fluxes, the initial fouling resistance α_i_ (for certain feed-fluid composition) can be correlated with flux J as in previous studies; i.e.,

(9) $$\alpha_{i}\;={\gamma J}^{\beta }$$

**Figure 6:**
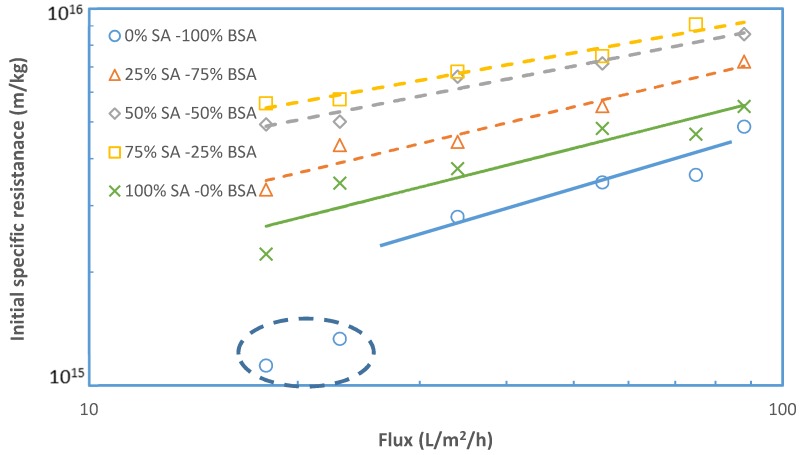
Initial fouling resistance α_i_ values with respect to permeate flux J.

Figure 6 depicts the initial resistance α_i_ data from this study, for the various feed fluid compositions. It is interesting that, with the exception of data at low fluxes for 100% BSA, all the other data appear to be correlated well with an exponent β ≈ 0.4. This value is relatively close to β values obtained in a previous study for polysaccharides [29]. The relatively small fouling resistance values obtained at low fluxes with 100% BSA foulants are difficult to explain and require additional work for clarification. The strong effect of flux on the initial fouling resistance α_i_ is attributed to the significant increase of concentration polarization (CP) with increasing flux and the increased drag forces on macro-molecular complexes; CP facilitates organic matter complexation and gel layer formation, whereas drag forces are expected to contribute to attachment of such complexes on the membrane and to enhance fouling layer coherence. As is evident in Figure 6, the value of factor γ (determining the level of α_i_) depends on the composition of the foulants.

**Figure 7:**
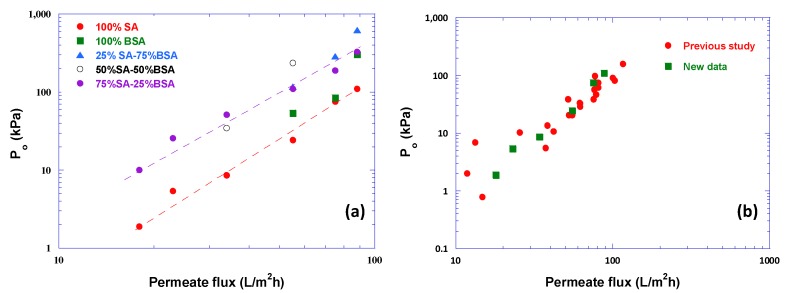
Values of parameter P_o_ (Equation (8)) versus permeate flux J. (**a**) Data from this study; (**b**) comparison of P_o_ data, for 100% SA, from this study with data from previous work [31].

In Figure 7a the parameter P_o_ data are plotted versus flux J, showing systemically increasing P_o_ values with increasing J. The two trend-lines drawn therein correspond to the cases 100% SA and 75% SA-25% BSA, that exhibit fairly strong compressibility effects. Correlation of P_o_ with flux J is considered useful, enhancing the archival value of these data and facilitating future modeling studies. Figure 7b shows very good agreement of P_o_ data for the case 100% SA with similar data from previous work [31].

**Figure 8:**
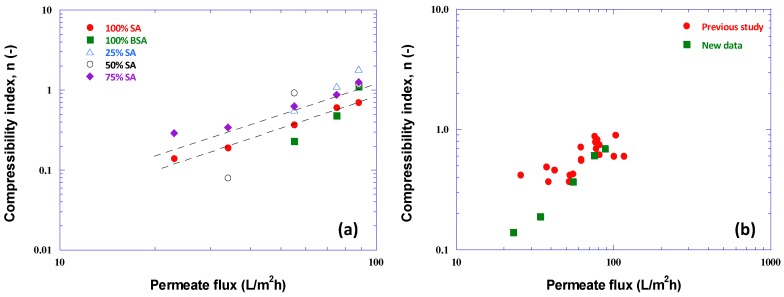
Compressibility index n values versus permeate flux. (**a**) Data from all tests with various foulant compositions; (**b**) comparison of data taken, with 100% SA foulants, in this and a previous study [31].

The trend of compressibility index n values versus flux J for the various cases, is similar to that of the preceding graphs. As shown by the trend lines, drawn in Figure 8a, for the cases 75% SA-25% BSA and 100% SA, the n values of foulant-mixtures exhibiting significant compressibility are well correlated with flux J. For other cases, very small or missing (zero) n-values indicate very small or no compressibility of the fouling layer; i.e., α = α_i_ = const. Figure 8b also shows rough agreement of the n values for 100% SA with respective values from a previous study [31]. Table A1 in Appendix A includes all the parameter values as well as the aforementioned correlations (with flux J) of α_i_, P_o_ and n.

### 4.4. Organic Matter Rejection

**Figure 9:**
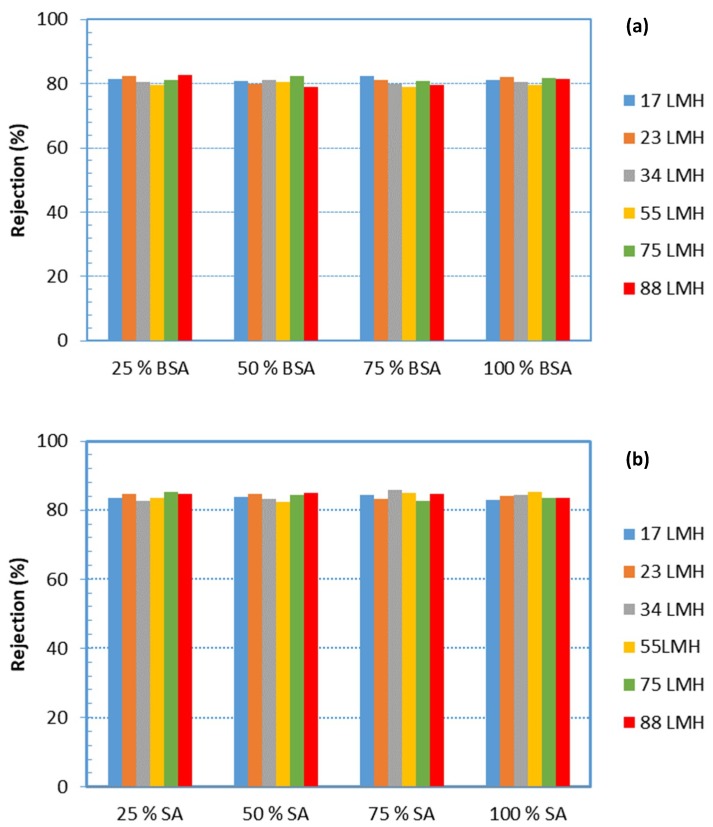
Rejection of organic matter by the UF membrane during filtration, for all cases. (**a**) Rejection of proteins; (**b**) rejection of polysaccharides.

Figure 9a,b show alginate and BSA rejection by the UF membrane, which is at the level of ~85% and ~80%, respectively. It is noted that these rejection values were obtained from measurements of the composite concentration of the respective species in permeate samples, collected over the entire time-period of testing, thus providing an average rejection over this sampling period. However, it is not expected that the instantaneous values would differ significantly from the mean values of Figure 9. The constant foulants rejection, over a relatively broad range of fluxes, suggests that the increasing fouling resistance with permeate flux is due to *fouling layer compressibility effects* and not due to pore constriction, as one might have assumed.

**Figure 10:**
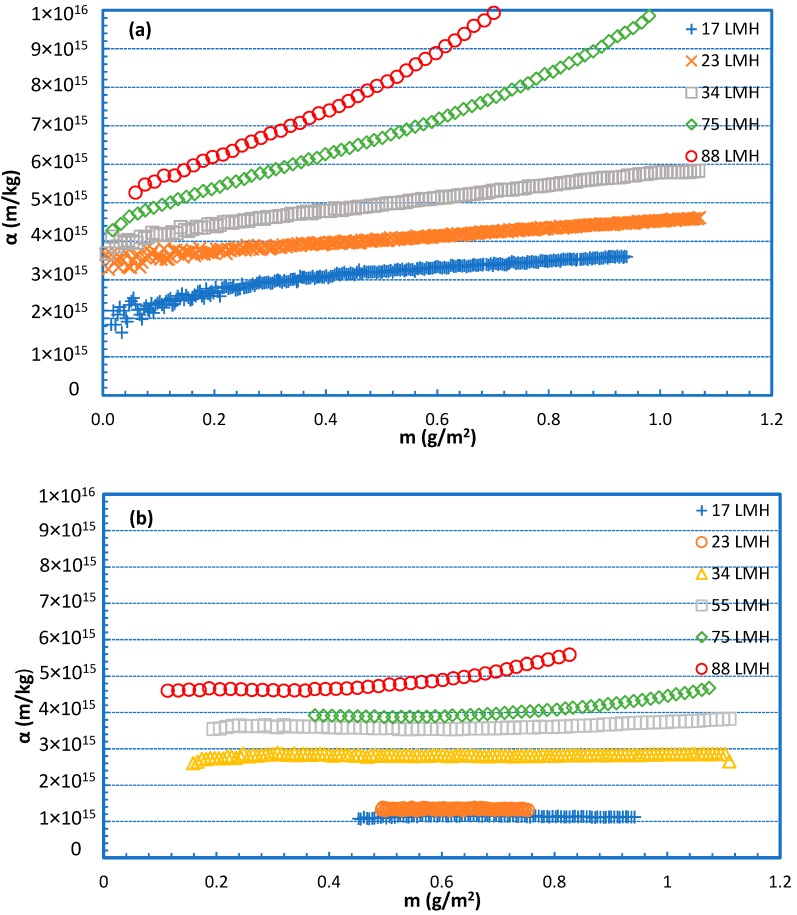
Specific fouling resistance α versus deposited foulant-mass density m. Fouling species: (**a**) 100% SA and (**b**) 100% BSA.

Interesting and useful information on the evolution of fouling resistance α can be also obtained by plotting α data versus the deposited foulant-mass density m (g/m^2^) on the membrane, as shown in Figure 10. Foulant density m is determined by considering total deposition of the *rejected organic mass* that corresponds to the permeate volume; the rejected mass is estimated by employing the data of Figure 9. The graphs in Figure 10 also show the increasing resistance α with deposited foulant mass, which is non-linear at the high fluxes; this trend apparently reflects structural changes of the fouling layer with increasing permeate drag, as also shown in other preceding graphs (Figure 6).

It should be added that in both Figure 9 and Figure 10, the mean fluxes at each level (i.e., 17 L/m^2^h, 23 L/m^2^h, 34 L/m^2^h, etc.) were marked; details on fluxes in each test are provided in the Appendix A Table A1.

**Figure 11:**
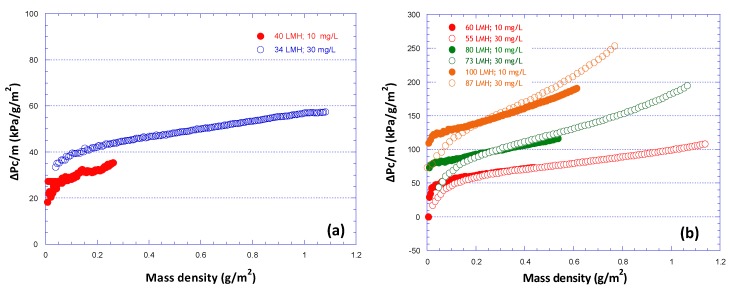
Variation of the quantity [ΔP_c_/m] with increasing deposit-mass density, for various permeate flux values; fouling species 100% SA. Foulants concentration: 30 mg/L, this study; 10 mg/L, previous study [31]. (**a**) Flux J = 40 L/m^2^h; (**b**) J = 60–100 L/m^2^h.

The quantity (ΔP_c_/m) plotted versus deposited mass density m, is also of interest in analyzing the data [29] as well as in future modeling studies; again, nonlinear variation is indicative of structural changes of an evolving fouling layer. In Figure 11, a comparison is shown of such data from the present and another study [31], for 100% SA foulants. Agreement of these data is very good for relatively high fluxes (Figure 11b), even though the foulants concentration was different in these experimental campaigns. The quantitative difference for flux 40 L/m^2^h (Figure 11a), between data corresponding to 30 mg/L (this study) and 10 mg/L [31] may be explained on the basis of the smaller concentration (and smaller mass rate of deposition) in the previous study that may have led to a different fouling layer structure, compared to filtration of higher concentration solution.

### 4.5. Effect of Feed-Solution Origin/Preparation on Fouling Resistance a

**Figure 12:**
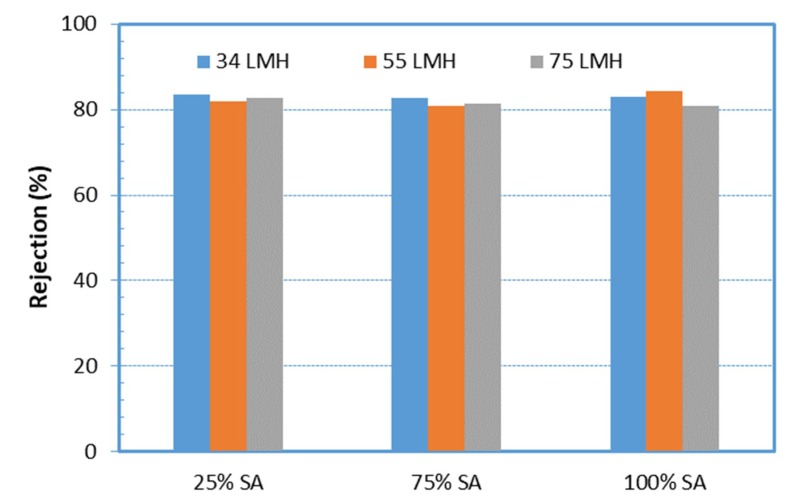
Protein rejection during filtration of feed-solutions, prepared by an alternative protocol, for various foulants composition and permeate fluxes.

**Figure 13:**
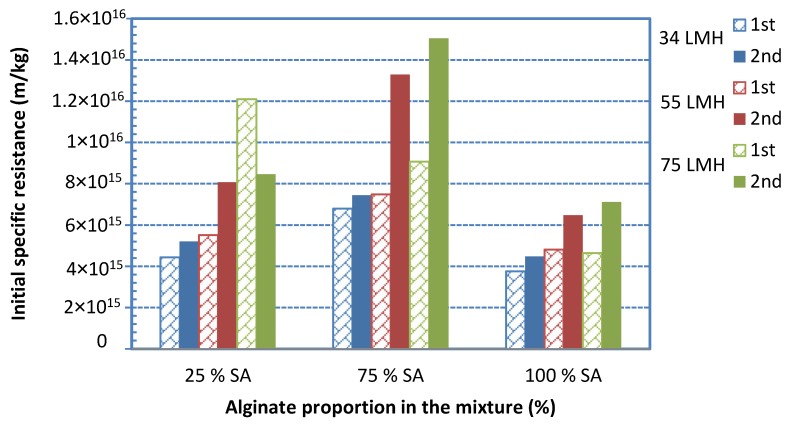
Initial fouling resistance α_i_ values for various foulant compositions and fluxes, with feed-fluid prepared by different protocols: “1^st^” and “2^nd^” means main and alternate protocol, respectively.

A limited number of tests was carried out with feed waters prepared under an alternative protocol, as described in preceding Section 3.2, in order to examine whether the protocol of the synthetic feed-water influenced the fouling behavior/propensity of the respective solution. Typical data from such experiments are included in Figure 12 and Figure 13. In the former, the protein rejection characteristics are shown for three fluxes and various compositions of feed-solution, prepared by the alternative (2nd) protocol. One observes that the protein rejection is almost identical to that obtained in filtration of solutions of the same composition, but prepared with a different protocol, depicted in Figure 9a. This agreement indicates some similarity of the two feed solutions, particularly in regard to small organic molecules that might penetrate the UF membrane.

Figure 13 shows data on initial fouling resistance α_i_ for various foulant compositions and fluxes, employing feed-fluid prepared by an alternative protocol. The same trends observed in tests with feed-fluid prepared by a different protocol (Figure 5) are exhibited here. Indeed, by increasing the percentage of SA (in mixtures with BSA), the initial fouling layer resistance α_i_ tends to significantly increase above that of either BSA or SA alone in solution. Again, the greatest α_i_ values were obtained with the 25% BSA-75% SA mixture for the three constant flux tests, as also observed in Figure 5. However, the magnitude of resistance α_i_ appears to be greater in the data of Figure 13, compared to those plotted in Figure 5 (except for the 25% SA mixture at a flux 75 L/m^2^h), which were taken with feed-fluid prepared by a different protocol. This difference may be attributed to a different mode of complexation of protein and polysaccharide molecules, due to the different sequence of mixing the various components, for final solution preparation.

## 5. Conclusions

The mechanism of fouling layer formation on the UF membrane appears to dominate in cases of feed-fluid comprising 100% SA and SA-BSA foulant-mixtures. In cases of foulants comprising 100% BSA, or mixtures rich in BSA, other mechanisms may be operative in the initial phase of fouling; i.e., pore blocking, and possibly constriction to a limited extent, for the particular PAN membrane employed. Beyond that period, the mechanism of fouling layer covering the membrane also prevails.

The effect of permeate flux is strong, particularly above ~30 L/m^2^h. For the BSA-SA mixtures studied, gel-type fouling layers appear to develop that exhibit significant compressibility at the higher permeate fluxes J. The strong compressibility effects are manifested in the nonlinear temporal increase of the pressure drop across the fouling layer, ΔP_c_, with increasing deposited organic mass. A suggestion of practical interest emerges from the new data with mixed BSA-SA organic foulants of UF membranes; i.e., one should restrict the range of operability of related UF operations to low fluxes.

The *specific fouling-layer resistance α* strongly depends on permeate flux J and on the composition of SA-BSA mixture in the feed-water. For the used brackish feed-water (with Ca^2+^), a strong SA-BSA interaction apparently occurs in the depositing layer, possibly mediated by Ca^2+^ ions; this leads to significantly greater fouling-layer resistance α in the case of SA-BSA mixtures, compared to either SA or BSA alone in the filtered feed-water. Feed-waters rich in SA, in proportion to BSA, exhibit the greatest membrane fouling resistance.

The initial fouling-layer resistance α_i_ is well-correlated with permeation flux J by an expression of the form α_i_ = γJ^β^, where the exponent for almost all cases studied is β ≈ 0.4 and the factor γ depends on the foulants composition. Furthermore, the evolution of fouling-layer resistance α can be correlated by a three-parameter expression that include α_i_, P_o_ and n. The latter two parameters are a reference pressure and an index accounting for compressibility effects, that tend to increase with flux J. These correlations are considered significant in that they enhance the archival value of data, and provide necessary basic relations for future modeling and development of useful simulation tools.

In the present study, useful results were obtained with flat-sheet UF membranes. However, it would be of practical interest to further investigate the case of mixed foulants in hollow fiber UF membranes, employed in numerous applications. Such studies should also include the commonly practiced phase of membrane backwashing.

## Supplementary Materials

The following are available online at http://www.mdpi.com/2077-0375/9/2/21/s1. Table S1: Reagents and concentrated solutions employed for test solution preparation; Figure S1: Distribution of intrinsic (clean water) UF membrane resistance, R_m_; Table S2: Key parameters of fouling tests; main protocol for solution preparation; Table S3: Key parameters of fouling tests; alternative protocol for solution preparation.

## Appendix A

**Table A1:** Correlation of UF fouling resistance α data; fitting parameters.

Fouling Species	Flux (L/m^2^h)	α_i_ (m/kg)	P_o_ (kPa)	n (-)	Correlations
**SA 100%**	18	2.23 × 10^15^	1.9	0.30	α_i_ = γ·J^β^ where
	22	3.44 × 10^15^	5.4	0.14	γ = 6.94 × 10^14^, β = 0.46
	36	3.76 × 10^15^	8.6	0.19	P_o_ = γ·J^β^ where
	55	4.81 × 10^15^	24.4	0.37	γ = 1.98 × 10^−3^, β = 1.26
	73	4.64 × 10^15^	75.3	0.61	n = γ·J^β^ where
	87	5.50 × 10^15^	110.2	0.70	γ = 2.48 × 10^−3^, β = 1.26
**SA 75%-BSA 25%**	18	5.60 × 10^15^	10.0	0.23	α_i_ = γ·J^β^
	23	5.74 × 10^15^	25.7	0.29	γ = 2.13 × 1015, β = 0.33
	34	6.80 × 10^15^	51.6	0.34	P_o_ = γ·J^β^,
	55	7.48 × 10^15^	110.0	0.63	γ = 3.95 × 10^−2^, β = 2.00
	71	9.07 × 10^15^	188.9	0.87	n = γ·J^β^
	87	2.56 × 10^1^^6^	327.2	1.26	γ = 8.37 × 10^−3^, β = 1.09
**SA 50%-BSA 50%**	17	4.93 × 10^15^	0	0	α_i_ = γ·J^β^
	24	5.01 × 10^15^	0	0	γ = 1.75 × 10^15^, β = 0.36
	37	6.60 × 10^15^	34.5	0.08	P_o_ = γ·J^β^,
	54	7.15 × 10^15^	235.6	0.92	γ = 1.04 × 10^−2^, β = 2.37
	88	8.55 × 10^15^	327.3	1.19	n = γ·J^β^
					γ = 84.96E-06, β = 2.85
**SA 25%-BSA 75%**	18	3.31 × 10^15^	0	0	α_i_ = γ·J^β^
	23	4.34 × 10^15^	0	0	γ = 9.89 × 10^14^, β = 0.44
	35	4.43 × 10^15^	0	0	P_o_ = γ·J^β^,
	56	5.51 × 10^15^	117.3	0.56	γ = 1.04 E-4, β = 3.47
	73	1.21 × 10^1^^6^	287.0	1.11	n = γ·J^β^
	89	7.23 × 10^15^	624.7	1.84	γ = 2.57 × 10^−5^, β = 2.47
**BSA 100%**	17	1.13 × 10^15^	0	0	α_i_ = γ·J^β^
	25	1.33 × 10^15^	0	0	γ = 4.87 × 10^14^, β = 0.48
	38	2.80 × 10^15^	0	0	P_o_ = γ·J^β^,
	57	3.46 × 10^15^	53.9	0.23	γ = 6.32 × 10^−5^, β = 3.37
	74	3.62 × 10^15^	84.5	0.48	n = γ·J^β^
	93	4.86 × 10^15^	302.8	1.11	γ = 5.44 × 10^−7^, β = 3.21
