# Supplementary material for: Membrane Fouling Due to Protein—Polysaccharide Mixtures in Dead-End Ultrafiltration; the Effect of Permeation Flux on Fouling Resistance
Source: Membranes (Basel). 2019 Feb 1;9(2):21. doi: 10.3390/membranes9020021 (PMC6409950; doi:10.3390/membranes9020021)
Supplement: Supplementary file 1 [file membranes-09-00021-s001.pdf]

SUPPLEMENT TO

Membrane Fouling Due to Protein – Polysaccharide Mixtures in Dead-End Ultrafiltration; the Effect of Permeation Flux on Fouling Resistance

Table S1. Reagents and concentrated solutions employed for test solution preparation.

Parameter	Concentrated Solution (g/L)	Volume Employed (mL)
Sodium alginate, SA	1.0	0–30
Bovine serum albumin, BSA	1.0	0–30
Sodium chloride, NaCl	75	20
Calcium chloride dihydrate, $CaCl_2{\cdot}2H_2O$	29.4	5
Tap water	0.5* (eq. NaCl)	945
Hydrochloric acid, HCl	37% (w/w)	–

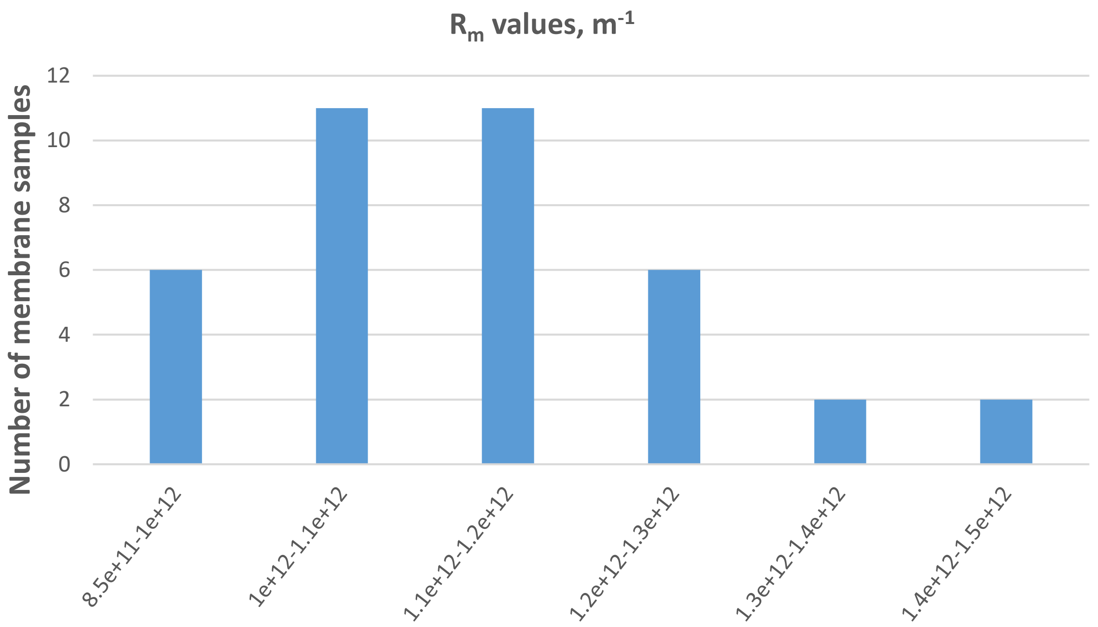

Figure S1. Distribution of intrinsic (clean water) UF membrane resistance, R_m.

Table S2. Key parameters of fouling tests; main protocol for solution preparation.

Fouling Species	Flux (L/m^{2}h)	Duration t_f (min)	Volume at t_f (mL)	ΔPc at t_f (kPa)	Rm (m^{-1})
SA (100%)	18	140	50	17.0	1.15×10^{12}
	22	135	57	30.0	1.03×10^{12}
	36	76	57	62.4	1.14×10^{12}
	55	53	60	125.0	1.09×10^{12}
	73	36	58	213.2	1.15×10^{12}
	87	23	42	200.0	1.25×10^{12}
SA (75%)-BSA (25%)	18	131	50	25.7	1.02×10^{12}
	23	120	56	40.0	9.45×10^{11}
	34	85	59	96.7	1.19×10^{12}
	55	48	55	208.1	1.07×10^{12}
	71	28	41	218.0	9.11×10^{11}
	87	17	10	157.2	1.13×10^{12}
SA (50%)-BSA (50%)	17	138	50	22.1	1.08×10^{12}
	24	120	57	36.6	9.17×10^{11}
	37	112	61	63.5	1.20×10^{12}
	54	52	58	207.1	1.08×10^{12}
	88	18	31	196.6	1.27×10^{12}
SA (25%)-BSA (75%)	18	134	50	19.2	8.98×10^{11}
	23	126	60	32.2	1.38×10^{12}
	35	82	58	49.8	1.18×10^{12}
	56	52	60	146.9	1.15×10^{12}
	73	35	24	209.1	1.04×10^{12}
	89	22	40	207.2	9.77×10^{11}
BSA (100%)	17	106	40	9.2	1.13×10^{12}
	25	95	50	12.4	1.05×10^{12}
	38	76	59	32.3	1.08×10^{12}
	57	50	60	70.2	1.17×10^{12}
	74	30	49	92.5	1.27×10^{12}
	93	30	57	196.7	1.21×10^{12}

Table S3. Key parameters of fouling tests; alternative protocol for solution preparation.

Fouling species	Flux (L/m^{2}h)	Duration t_f (min)	Volume at t_f (mL)	ΔPc at t_f (kPa)	Rm (m^{-1})
SA (100%)	35	82	60	60.9	1.04×10^{12}
	55	53	61	130.3	1.04×10^{12}
	69	31	46	194.9	1.44×10^{12}
SA (75%)-BSA (25%)	33	84	59	93.6	1.23×10^{12}
	52	42	46	237.6	8.57×10^{11}
	61	25	38	351.4	1.34×10^{12}
SA (25%)-BSA (75%)	33	86	61	60.1	1.17×10^{12}
	52	54	60	199.0	1.56×10^{12}
	71	51	33	236.9	1.26×10^{12}
